# Chondrogenic differentiation induced by extracellular vesicles bound to a nanofibrous substrate

**DOI:** 10.1038/s41536-021-00190-8

**Published:** 2021-11-19

**Authors:** Marta R. Casanova, Hugo Osório, Rui L. Reis, Albino Martins, Nuno M. Neves

**Affiliations:** 1 3B’s Research Group, I3Bs - Research Institute on Biomaterials, Biodegradables and Biomimetics of University of Minho, Headquarters of the European Institute of Excellence on Tissue Engineering and Regenerative Medicine, AvePark – Parque de Ciência e Tecnologia, Zona Industrial da Gandra, 4805-017 Barco/Guimarães, Portugal; 2 ICVS/3B’s – PT Government Associate Laboratory, Braga/Guimarães, Portugal; 3 i3S — Instituto de Investigação e Inovação em Saúde, Universidade do Porto, 4200-135 Porto, Portugal; 4 Ipatimup—Institute of Molecular Pathology and Immunology of the University of Porto, University of Porto, 4200-135 Porto, Portugal; 5 Department of Pathology, Faculty of Medicine, University of Porto, 4200-319 Porto, Portugal

**Keywords:** Stem-cell differentiation, Biomaterials - cells, Mesenchymal stem cells

## Abstract

Extracellular vesicles (EVs) are being increasingly studied owing to its regenerative potential, namely EVs derived from human bone marrow mesenchymal stem cells (hBM-MSCs). Those can be used for controlling inflammation, repairing injury, and enhancing tissue regeneration. Differently, the potential of EVs derived from human articular chondrocytes (hACs) to promote cartilage regeneration has not been thoroughly investigated. This work aims to develop an EVs immobilization system capable of selectively bind EVs present in conditioned medium obtained from cultures of hACs or hBM-MSC. For that, an anti-CD63 antibody was immobilized at the surface of an activated and functionalized electrospun nanofibrous mesh. The chondrogenic potential of bound EVs was further assessed by culturing hBM-MSCs during 28 days under basal conditions. EVs derived from hACs cultured under differentiation medium or from chondrogenically committed hBM-MSCs induced a chondrogenic phenotype characterized by marked induction of *SOX9*, *COMP*, *Aggrecan* and *Collagen type II*, and matrix glycosaminoglycans synthesis. Indeed, both EVs immobilization systems outperformed the currently used chondroinductive strategies. These data show that naturally secreted EVs can guide the chondrogenic commitment of hBM-MSCs in the absence of any other chemical or genetic chondrogenic inductors based in medium supplementation.

## Introduction

Human articular cartilage is composed of an extensive extracellular matrix (ECM) with a sparse distribution of specialized cells, the chondrocytes. The chondrocytes are responsible to maintain the cartilage matrix structure, composition and properties. In adults, articular cartilage has limited or no potential of self-repair; thus, damaged cartilage needs surgical interventions for either repair or replacement of the joint^1,2^. The available treatments are based on the transplantation of autologous cells (e.g., autologous chondrocyte implantation (ACI))^3,4^ and ex vivo engineered tissue implants (matrix-induced ACI (MACI))^5–7^.

Human bone marrow mesenchymal stem cells (hBM-MSCs) are attractive candidates for advanced cell therapies, including cartilage regeneration^8^. hBM-MSCs can be induced to differentiate into the chondrogenic lineage when exposed to specific cocktails of growth factors^8–10^. The difficulty in obtaining a well-defined population and maintain a stable cartilaginous phenotype of the differentiated MSCs, preventing them from progressing toward osteogenesis^11^, leads to the investigation of approaches beyond the standard chondrogenic medium^12,13^.

Extracellular vesicles (EVs) are lipidic particles (exosomes with 30–100 nm or microvesicles with 50–2000 nm diamater) secreted by cells which deliver biological signals, namely proteins, lipids and nucleic acids (DNA, mRNA and microRNA and tRNAs), to target cells, protecting them during traveling^14–18^. EVs can be isolated from virtually all biological fluids, namely blood, saliva, urine, synovial fluid, pleural effusions, ocular effluent, aqueous humor, nasal secretions, breast milk, amniotic fluid, cerebrospinal fluid, bile and semen^19^. The major biomarkers related to EV biogenesis include the tetraspanins (CD63, CD81, and CD9), ALIX and TSG101. Among these, the transmembrane protein CD63 is usually used as a representative exosomal protein marker^20^.

The regenerative potential of EVs has been described for a wide range of tissues, including the heart and blood vessels, kidney, liver, lung, skin, neural, and reproductive tissue^21–23^. Among the different types, MSCs are one of the most widely used cell source for generating EVs^24–27^. It is believed that the MSC-derived EVs share the same anti-inflammatory and trophic properties of the parental MSCs, exerting their therapeutic effects^24,26–28^. Indeed, EVs are being recognized by its regenerative potential for controlling inflammation, repairing injury, and enhancing tissue regeneration^18,21,28^. However, their potential in promoting cartilage repair and slowing degeneration has not been thoroughly investigated^29,30^. Moreover, the knowledge on the cartilage regenerative potential of EVs derived from hBM-MSCs or even from human articular chondrocytes (hACs) is very scarce in the scientific literature.

Envisioning an EVs immobilization system, we herein report the development of a nanofibrous mesh (NFM) functionalized with an anti-CD63 antibody able to specifically bind EVs derived from hACs or hBM-MSCs. We hypothesized that the EVs derived from hACs or chondrogenically differentiated hBM-MSCs can induce the chondrogenic commitment of homotypic cells. The regenerative potential of these EVs immobilization systems were investigated by assessing their capability to induce the chondrogenic differentiation of hBM-MSCs without the need of any further medium supplementation.

## Results

### Development and characterization of the EV immobilization systems

**Fig. 1:**
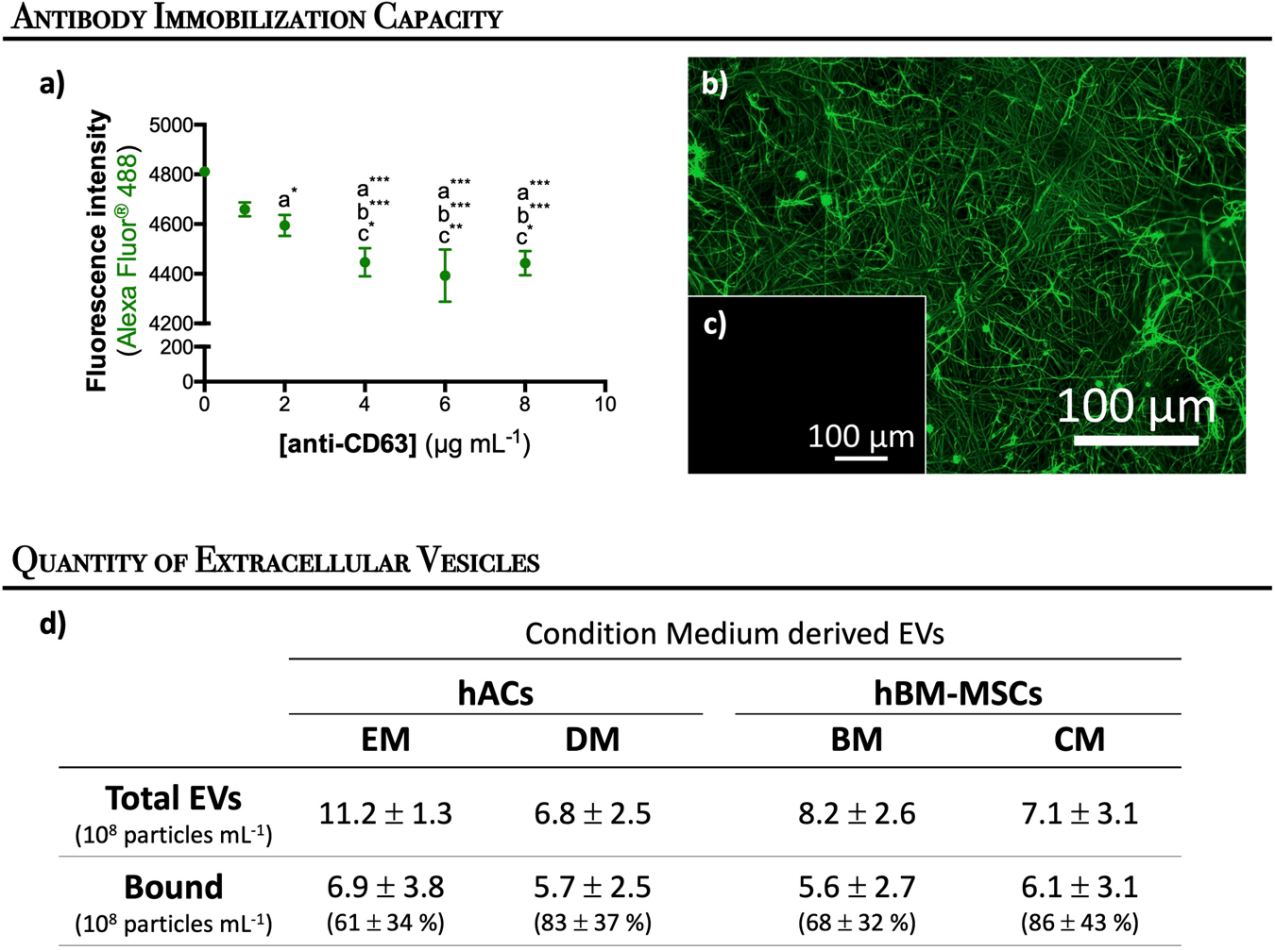
Antibody immobilization and extracellular vesicle binding capacity. Immobilization capacity of anti-CD63 antibody at the surface of activated and functionalized nanofibrous substrates (**a**). The data are represented as mean ± standard deviation and analyzed by one-way ANOVA test, followed by the Tukey’s HSD test (*p < 0.01; **p < 0.001; ***p < 0.0001): a denotes significant differences compared to concentration 0 μg mL^−1^; b denotes significant differences compared to concentration 1 μg mL^−1^, and c denotes significant differences compared to concentration 2 μg mL^−1^. Spatial distribution of anti-CD63 immobilized at the surface of activated and functionalized nanofibrous substrates at 4 μg mL^−1^ (**b**). The negative control sample was not incubated with the primary antibody (**c**). Quantification of extracellular vesicles derived from hACs and hBM-MSCs and bound to the biofunctional nanofibrous system (**d**).

To develop an immobilization system capable to selectively bind EVs from conditioned medium, a method to generate functional groups at the surface of electrospun polycaprolactone (PCL) NFMs was implemented, which provide binding sites for the biomolecule immobilization. Specifically, an anti-CD63 antibody was immobilized at the surface of activated and functionalized nanofibrous substrate capable to covalently and effectively bind EVs. In order to determine the maximum immobilization capacity of the system, a wide range of anti-CD63 antibody concentrations (0–8 μg mL^−1^) were used. According to an indirect quantification method, the maximum antibody immobilization was achived approximately at the concentration of 4 μg mL^−1^ (Fig. 1a). A uniform distribution of anti-CD63 antibody immobilized at the surface of the nanofibrous substrate can be observed by fluorescence microscopy (Fig. 1b).

The EVs’ binding capacity of the immobilized anti-CD63 antibody was assessed by using conditioned media harvested from hACs cultured under expansion or differentiation media, as well as from hBM-MSCs cultured under basal or chondrogenic media (i.e., AC^EM^, AC^DM^, MSC^BM^, and MSC^CM^, respectively). The biological status of the EVs’ donor cells is reported in Supplementary Fig. 1. Figure 1d shows the range of EVs’ concentrations obtained from the four different conditioned media (from 6.8 to 11.2 × 10^8^ particles mL^−1^). Those EVs were successfully bound at the surface of the biofunctional nanofibrous substrate on the range of 5.6–6.9 × 10^8^ particles mL^−1^.

**Fig. 2:**
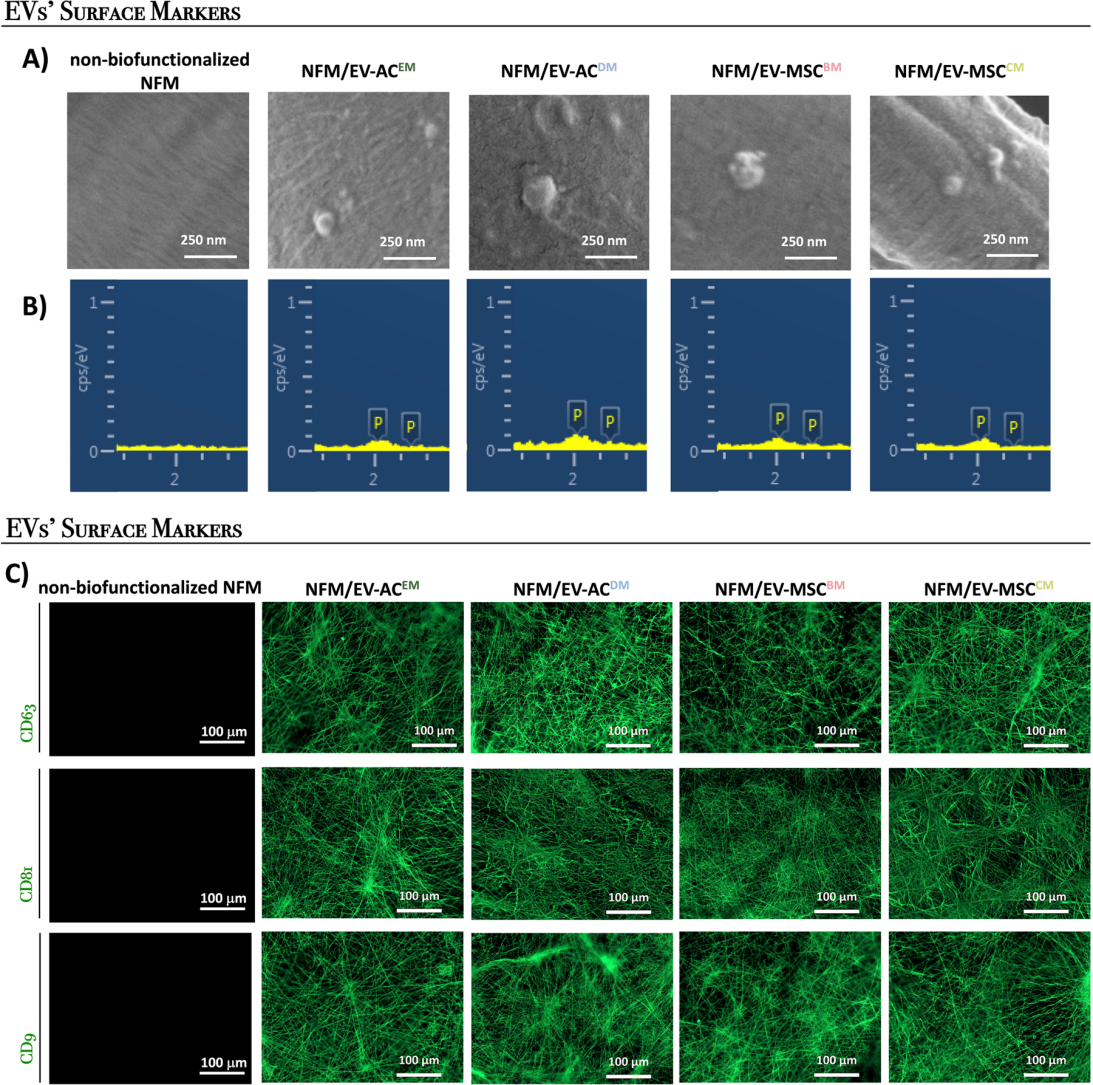
Distribution of bound extracellular vesicles. Distribution of EVs bound at the surface of the biofunctional nanofibrous system (**A**); EDS spectrum of the EVs immobilization system surface (**B**); fluorescence micrographs of the EVs markers CD63, CD81, and CD9 (**C**).

Figure 2 presents the distribution of EVs bound at the surface of the nanofibrous substrate. In the scanning electron microscopic (SEM) micrographs (Fig. 2A), bound EVs with diameters of approximately 100 nm can be identified (Supplementary Table 1). Their presence can be also confirmed by the energy-dispersive spectroscopic (EDS), namely by the presence of the P element from the phospholipids of EVs (Fig. 2B). To ascertain about the phenotypic profile of bound EVs, the expression of the tetraspanins CD63/CD81/CD9 was analyzed by fluorescence microscopy (Fig. 2C), showing a uniform distribution at the surface of the biofunctional nanofibrous substrate.

**Fig. 3:**
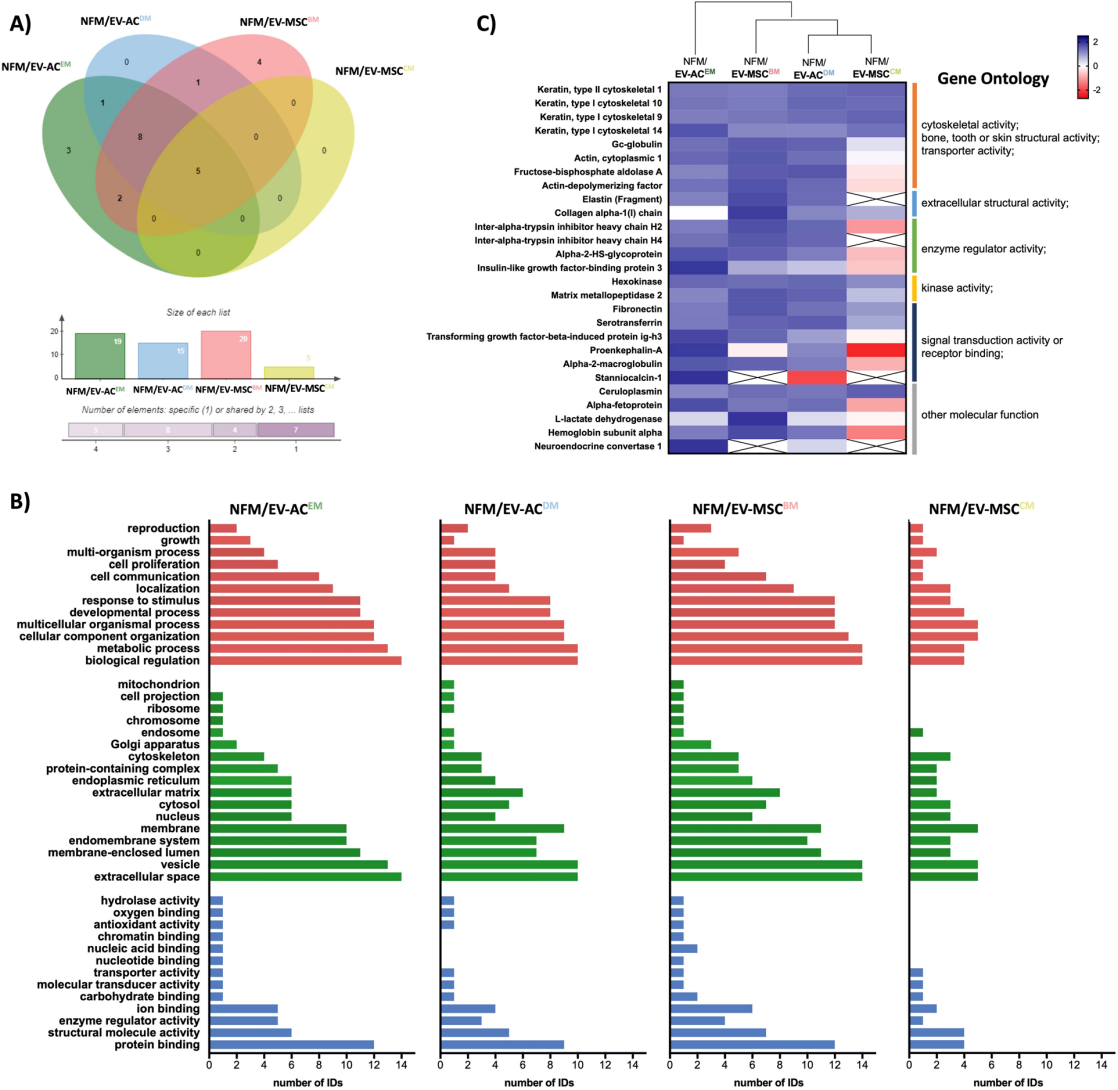
Bioinformatic analysis of the protein content of EVs derived from different source (i.e., hACs under expansion medium [*NFM/EV-AC*^*EM*^], hACs under differentiation medium [*NFM/EV-AC*^*DM*^], hBM-MSCs under basal medium [*NFM/EV-MSC*^*BM*^], and hBM-MSCs under chondrogenic medium [*NFM/EV-MSC*^*CM*^]). Venn diagrams (**A**); Gene Ontology (GO) analysis (red: biological process, green: cellular component, blue: molecular function) (**B**); and heat map (clusters are assembled by GO analysis using a distance function of Euclidean) (**C**).

A detailed analysis of the protein content of the different EVs’ sources revealed 10, 15, 20, and 5 proteins in the content of *NFM/EV-AC*^*EM*^, *NFM/EV-AC*^*DM*^, *NFM/EV-MSC*^*BM*^, and *NFM/EV-MSC*^*CM*^ systems, respectively. The Venn diagram analysis revealed five common proteins, and three and four unique proteins on the *NFM/EV-AC*^*EM*^ and *NFM/EV-MSC*^*BM*^ systems, respectively (Fig. 3A). Gene Ontology (GO) analysis (Fig. 3B) showed that the molecular function (in blue) of those proteins are related to protein binding, structural molecule activity and enzyme regulator activity. Moreover, those proteins were mainly involved in biological processes (in red) such as biological regulation and metabolic process. In what concerns the cellular component (in green), the identified proteins are related to the extracellular space, as well as vesicles. Analyzing the expression of the different proteins in a heat map (Fig. 3C), it was possible to observe a downregulation of most of them in the *NFM/EV-MSC*^*CM*^ system, although clustered with the NFM/EV-MSC^DM^ system still. Also, the most related condition was the *NFM/EV-AC*^*EM*^.

### EV immobilization systems elicit hBM-MSC chondrogenic commitment

**Fig. 4:**
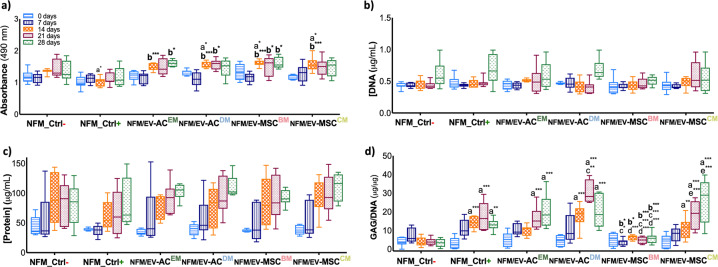
Biochemical performance of cultured hBM-MSCs. Metabolic activity (**a**), proliferation (DNA content) (**b**), total protein synthesis (**c**), and sulfated glycosaminoglycan (GAGs) content normalized against DNA (**d**) of hBM-MSCs cultured on biofunctional nanofibrous systems comprising EVs derived from different sources (i.e., *NFM/EV-AC*^*EM*^, *NFM/EV-AC*^*DM*^, *NFM/EV-MSC*^*BM*^, and *NFM/EV-MSC*^*CM*^), under basal culture conditions. hBM-MSCs cultured on non-biofunctionalized nanofibrous substrates under basal medium (*NFM_Ctrl*−) or chondrogenic medium (*NFM_Ctrl*+) were used as controls. Data are represented in box plot and analyzed by Kruskal–Wallis test, followed by the Tukey’s HSD test (*p < 0.01; **p < 0.001; ***p < 0.0001): a denotes significant differences compared to *NFM_Ctrl−*; b denotes significant differences compared to *NFM_Ctrl*+; c denotes significant differences *c*ompared to *NFM/EV-AC*^*EM*^; d denotes significant differences compared to *NFM/EV-AC*^*DM*^; e denotes significant differences compared to *NFM/EV-MSC*^*BM*^.

The ability of the EVs immobilization systems (*NFM/EV-AC*^*EM*^, *NFM/EV-AC*^*DM*^, *NFM/EV-MSC*^*BM*^, *NFM/EV-MSC*^*CM*^) to promote the onset of chondrogenesis of uncommitted homotypic cells was examined. The differentiation process is known to impair cell proliferation, due to an increase in the length of the cell cycle^31^, and to induce changes in the protein synthesis rate^32^. Therefore, we evaluated the biochemical profile of hBM-MSC metabolism, proliferation, and total protein synthesis. In terms of metabolic activity (Fig. 4a), on the 14^th^ day of culture, the hBM-MSCs cultured on the EVs immobilization systems (*NFM/EV-AC*^*EM*^, *NFM/EV-AC*^*DM*^, *NFM/EV-MSC*^*BM*^, *NFM/EV-MSC*^*CM*^) displayed significantly higher metabolism than the hBM-MSC control conditions (NFM_Ctrl+ and NFM_Ctrl−) (*p* < 0.0001). On the 28^th^ day of culture, the hBM-MSC culture on *NFM/EV-AC*^*EM*^ and *NFM/EV-MSC*^*BM*^ immobilization systems presented significantly higher metabolic activity than hBM-MSCs cultured in CM (*NFM_Ctrl*+) (*p* < 0.01). The EVs immobilization systems were favorable for cell proliferation (Fig. 4b) and protein synthesis (Fig. 4c), since their levels are comparable to those observed on the control culture conditions over time.

The chondrogenic inductive potential of bound EVs was then evaluated in terms of the extracellular matrix glycosaminoglycans (GAG) content (Fig. 4d). Along the time, the hBM-MSCs cultured under standard chondrogenic medium (*NFM_Ctrl*+) and EVs immobilization systems, namely the *NFM/EV-AC*^*EM*^, *NFM/EV-AC*^*DM*^ and *NFM/EV-MSC*^*CM*^, displayed a significantly higher GAG synthesis than the hBM-MSCs cultured under basal medium (BM) (*NFM_Ctrl−*) and the *NFM/EV-MSC*^*BM*^ system. On the 21^st^ day of hBM-MSC culture, the *NFM/EV-AC*^*DM*^ system presented significantly high GAG content when compared to the *NFM/EV-AC*^*EM*^ system (*p* < 0.01).

**Fig. 5:**
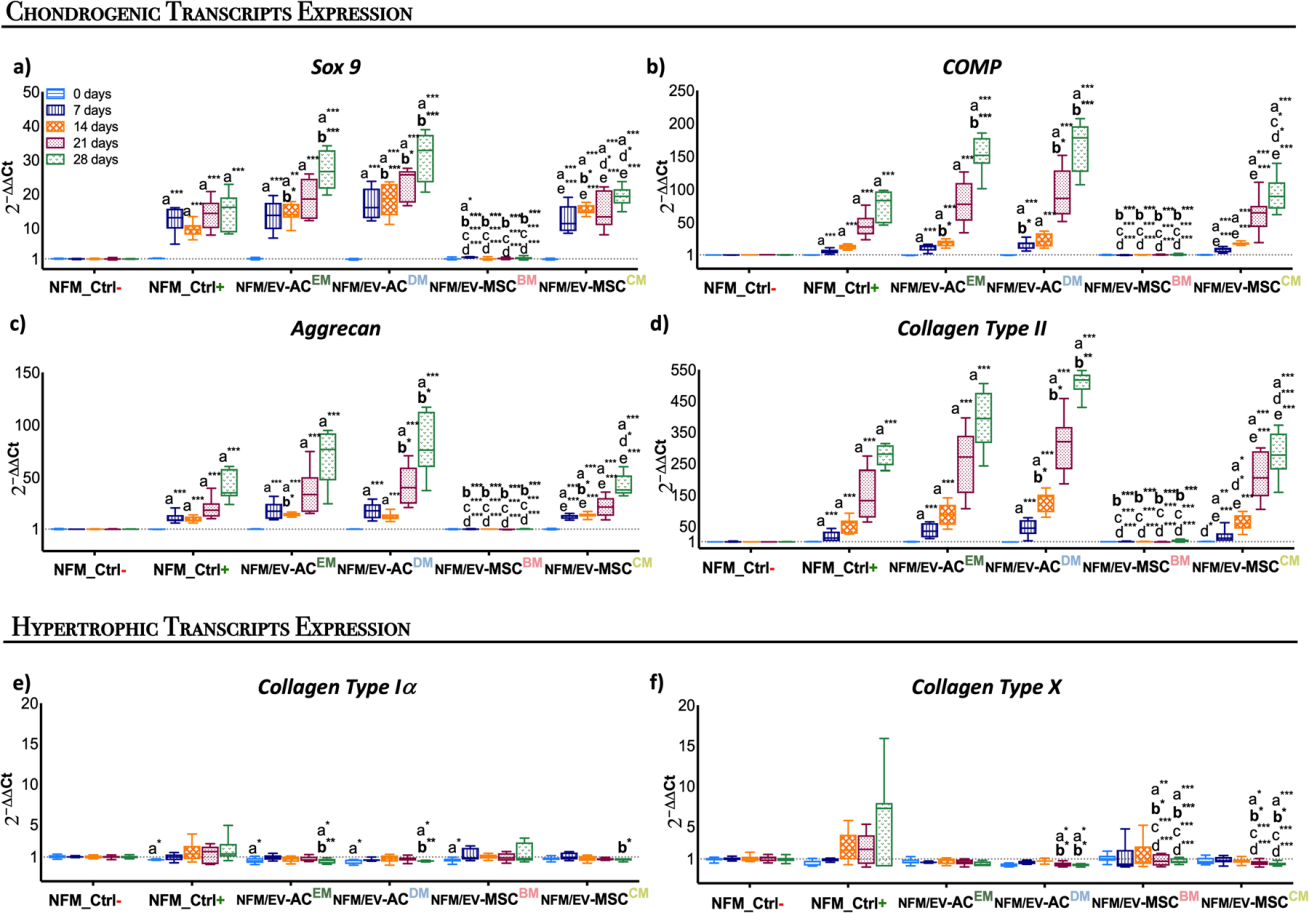
Temporal gene expression of cultured hBM-MSCs. Relative expression of chondrogenic (i.e., *Sox9* (**a**), *COMP* (**b**), *Aggrecan* (**c**), and *Collagen type II* (**d**)) and hypertrophic (i.e., *Collagen type Iα* (**e**), *Collagen type X* (**f**)) transcripts by hBM-MSCs cultured on biofunctional nanofibrous systems comprising EVs derived from different sources (i.e., *NFM/EV-AC*^*EM*^, *NFM/EV-AC*^*DM*^, *NFM/EV-MSC*^*BM*^, and *NFM/EV-MSC*^*CM*^), under basal culture conditions. hBM-MSCs cultured on non-biofunctionalized nanofibrous substrates under basal medium (*NFM_Ctrl-*) or chondrogenic medium (*NFM_Ctrl*+) were used as controls. Data are represented in box plot and analyzed by Kruskal–Wallis test, followed by Tukey’s HSD test (*p < 0.01; **p < 0.001; ***p < 0.0001): a denotes significant differences compared to *NFM_Ctrl-*; b denotes significant differences compared to *NFM_Ctrl*+; c denotes significant differences *c*ompared to *NFM/EV-AC*^*EM*^; d denotes significant differences compared to *NFM/EV-AC*^*DM*^; e denotes significant differences compared to *NFM/EV-MSC*^*BM*^.

To better define the chondrogenic commitment of the hBM-MSCs induced by the EVs immobilization systems, the temporal gene expression of chondrogenic markers was investigated (Fig. 5). The cartilage-related genes, namely *Sox9*, *COMP*, *Aggrecan*, and *Collagen type II*, are all overexpressed in all testing conditions, except on the *NFM/EV-MSC*^*BM*^ system. Along culture time, a significantly higher cartilage-related gene expression was observed on the EVs immobilization systems (*NFM/EV-AC*^*EM*^, *NFM/EV-AC*^*DM*^, *NFM/EV-MSC*^*BM*^, *NFM/EV-MSC*^*CM*^) (*p* < 0.01). On the 28^th^ day of culture, a significantly higher *Sox9* and *COMP* expression was observed in *NFM/EV-AC*^*EM*^ and *NFM/EV-AC*^*DM*^ systems when compared to the non-biofunctionalized NFMs under standard chondrogenic differentiation medium (DM) (*NFM_Ctrl*+) (*p* < 0.0001). Likewise, the *NFM/EV-AC*^*DM*^ system induced a significantly higher *Aggrecan* and *Collagen type II* expression than hBM-MSCs cultured under standard chondrogenic DM (*NFM_Ctrl*+) and the *NFM/EV-MSC*^*CM*^ system (*p* < 0.01). These results show that bound EVs, obtained during hBM-MSC chondrogenic differentiation and from hACs cultured under DM, provides guidance for chondrogenic lineage progression of homotypic cells. Therefore, it is also crucial to confirm that the hBM-MSCs were not undergoing hypertrophy and further differentiating into the osteogenic lineage. The lower hypertrophy-related genes (i.e., *Collagen type X*, *Collagen type Iα*) expression patterns were considerably similar in all the EVs immobilization systems, inversely to the standard chondrogenic DM (Fig. 5e, f). Moreover, those systems had significantly lower expression of the hypertrophy-related genes when compared with the non-biofunctionalized NFMs under chondrogenic media (*NFM_Ctrl*+) along the culture time.

**Fig. 6:**
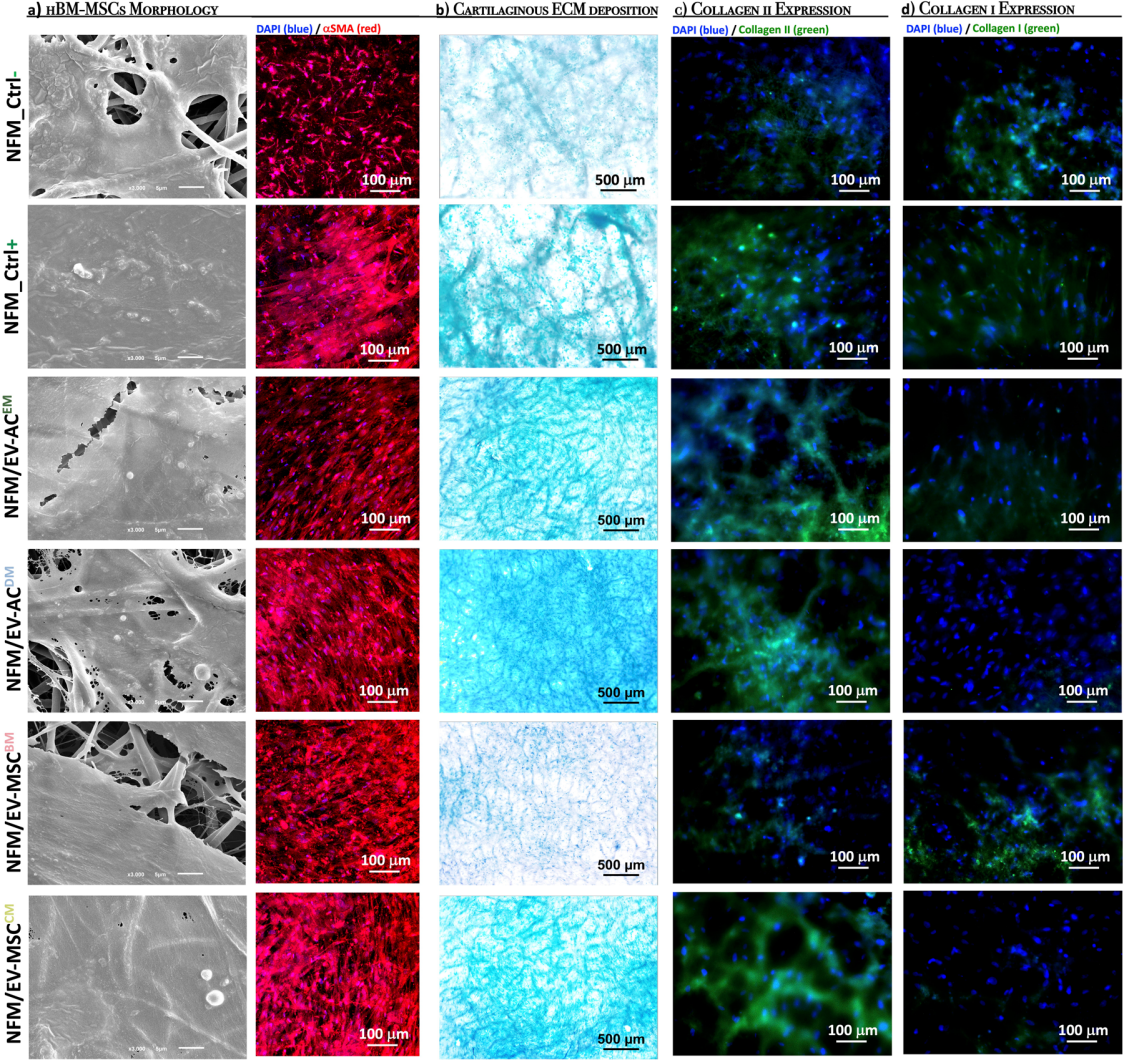
Morphology and extracellular matrix of cultured hBM-MSCs. Morphology of hBM-MSCs analyzed by scanning electron microscopy (SEM) and by immunofluorescence (red for actin and blue for nuclei) (**a**); Alcian blue staining of sulfated glycosaminoglycans (**b**); and immunoexpression of collagen type II (in green) (**c**) and type Iα (in green) (**d**); nuclei in blue. hBM-MSCs were cultured during 28 days on biofunctional nanofibrous systems comprising EVs derived from different sources (i.e., *NFM/EV-AC*^*EM*^, *NFM/EV-AC*^*DM*^, *NFM/EV-MSC*^*BM*^, and *NFM/EV-MSC*^*CM*^) under basal culture conditions. hBM-MSCs cultured on non-biofunctionalized nanofibrous substrates under basal medium (*NFM_Ctrl−*) or chondrogenic medium (*NFM_Ctrl*+) were used as controls.

The morphological changes of hBM-MSCs cultured on the different EVs immobilization systems reflect their differentiation stage (Fig. 6a). Alcian blue staining (Fig. 6b) confirms the presence of sulfated extracellular proteoglycans synthetized by hBM-MSCs cultured for 28 days on the EVs immobilization systems (*NFM/EV-AC*^*EM*^, *NFM/EV-AC*^*DM*^, *NFM/EV-MSC*^*CM*^), being more evident in the *NFM/EV-AC*^*DM*^ system. It is also possible to observe the absence of proteoglycan staining on the *NFM/EV-MSC*^*BM*^ system. Those results are corroborated by the immunoexpression of Collagen type II, which confirms the deposition of a cartilaginous ECM on the EVs immobilization systems (*NFM/EV-AC*^*EM*^, *NFM/EV-AC*^*DM*^, *NFM/EV-MSC*^*CM*^), accompanied by the absence of Collagen type Iα expression. These phenotypic observations were consistent with previously obtained results for GAG synthesis, as well as for the expression of cartilage-related genes.

## Discussion

EVs are small membrane-enclosed particles actively released by many cell types, including immune cells (T cells, B cells, dendritic cells, neutrophils, platelets), connective tissue cells (epithelial cells, fibroblasts), other specialized cells (endothelial cells, neuronal cells, chondrocyte cells), and stem cells^33,34^. Their prominent role in joint development and in the regulation of intra-articular homeostasis leads to recognize EVs as potential biomarkers of joint disease^21,23,35^. Therefore, they have been seen as a new tool to restore joint homeostasis and enhance articular cartilage regeneration, since they provide a simpler and safer alternative to current cell-based therapeutic options^27,28,30^. MSCs are one of the most prominent cell sources of EVs, and it is thought that the MSC-derived EVs mirror the content and fate of parent cells. Furthermore, EVs derived from MSC have been demonstrated to have positive effects over cell metabolism and proliferation^36,37^, angiogenesis^24,38^, and immunomodulation^39,40^ in a wide range of physiological systems. For example, in a clinical trial using MSC-derived EVs as treatment, symptoms were considerably mitigated in a patient with therapy-refractory graft-versus-host disease^41^.

Our experimental data showed that the culture conditions do not affect the yield of EVs (Fig. 1d). Furthermore, we showed that our biofunctional nanofibrous substrate with immobilized anti-CD63 antibody was able to bound those cell-derived EVs at the range of 5.6–6.9 × 10^8^ EVs mL^−1^, being the EVs’ binding capacity of the system determined according to the EVs concentration presented on the different conditioned medium (AC^EM^, AC^DM^, MSC^BM^, and MSC^CM^). Consistent with MSCs expressing CD63^42^, CD81^43^, and CD9^44^, bound EVs also showed the presence of these surface markers, suggesting that they are derived from endocytosed lipid rafts^45^. Therefore, the parent cell source could affect the EVs’ surface proteins, their glycosylation or lipid composition, and their cargo.

The role of MSC-derived EVs as modulators of joint homeostasis suggested that an EVs immobilization system may be an interesting therapeutic alternative in cartilage repair. The developed EVs immobilization systems comprising EVs derived from differentiated hACs or chondrogenically committed hBM-MSCs outperformed the effect of current chondroinductive strategies, in terms of the type and/or intensity of the signals. Specifically, the early activation of key chondrogenic commitment genes such as *Sox9* and *Aggrecan*, are considered necessary and sufficient to induce cartilage formation^46–49^. In addition, EVs immobilization systems (*NFM/EV-AC*^*EM*^, *NFM/EV-AC*^*DM*^, *NFM/EV-MSC*^*CM*^) induced a transient upregulation of downstream matrix-associated genes and proteins that may be required to promote cell–cell and cell–matrix interactions and support long-term differentiation, maintaining a stable cartilage phenotype of the differentiated MSCs^11^. However, an in vivo study is necessary to fully validate this strategy and confirm that those bound EVs (*NFM/EV-AC*^*EM*^, *NFM/EV-AC*^*DM*^, *NFM/EV-MSC*^*CM*^) are effective in promoting cartilage regeneration.

Notably, slight differences were noted between the three EVs immobilization systems (*NFM/EV-AC*^*EM*^, *NFM/EV-AC*^*DM*^, *NFM/EV-MSC*^*CM*^), suggesting that the cargo of EVs derived from hACs under DM (*NFM/EV-AC*^*DM*^) may also contributed to a stronger effect. Indeed, these observations were corroborated by the bioinformatics analysis of the EVs’ protein content. Articular cartilage vesicles containing RNA can be transferred into chondrocytes leading to a phenotypic change^50^. Indeed, it is reported that EVs are being constitutively released by normal articular chondrocytes^51^, participating in non-classical protein secretion, intercellular communication, and pathologic calcification^52,53^. In order to protect nearby chondrocytes from damage, articular chondrocyte vesicles provide extracellular matrix repair in pericellular cartilage and act to sequester substances such as ATP, calcium, and phosphate in toxic concentrations^50,52,54^.

Cell-derived EVs have similar biological functions to the parent cells, presenting several advantages due to their small size, low immunogenicity, and depletion of the common issues associated with direct cell injection. However, achieving an effective and controlled delivery of EVs at the target site is challenging, but is paramount to the efficient restoration of joint homeostasis and sustained effect on the regenerative process. Therefore, the use of our EVs immobilization system is expected to be more effective than currently available therapeutics that use soluble proteins or RNA molecules, which are usually prone to fast degradation after injection^55^. Using the herein presented strategy, we can promote a local delivery of bioactive molecules able to control the cellular activity, with more prolonged effect due to the binding of EVs at the surface of biofunctional nanofibrous substrates, avoiding degradation.

Envisioning the clinical translation of the different EVs immobilization systems herein reported, we foresee their potential use as an autologous cartilage regeneration strategy. For example, in the microfracture approach, the EVs immobilization system can be implanted into the defect site, where uncommitted homotypic cells migrating from the microperforations could be induced to differentiate into the chondrogenic lineage by the bound EVs. In an ex vivo attempt, both the ACs/MSCs and the bioactive factors (e.g., EVs) isolated from the same patient can be cultured on a biofunctional nanofibrous substrate, which will be then implanted into the defect site as a MACI approach. Both methods could induce the regeneration of cartilage, envisioning a personalized treatment of cartilage injuries.

This work shows the beneficial effect of cell-secreted factors, namely, EVs secreted by hACs and hBM-MSCs, playing an important role in the modulation of cell fate. Those EVs were successfully bound at the surface of biofunctional nanofibrous substrates, immobilizing the anti-CD63 antibody. The EVs immobilization systems comprising EVs derived from differentiated hACs and chondrogenically committed hBM-MSCs successfully induce the chondrogenic differentiation of homotypic cells more efficiently than the current chondroinductive strategies. Therefore, EVs immobilization systems are potential highly effective cell-free/secretome-based therapies for cartilage repair.

## Methods

### Isolation and expansion of hACs

Human cartilage samples were collected under Informed Consent from patients undergoing knee arthroplasty (female; aged 69 years) in the Hospital Center of Alto Ave, Guimarães, Portugal, in accordance with the established Protocol (67/CA). After the surgeries, a small part of the non-compromised knee cartilage was sectioned and preserved in phosphate-buffered saline (PBS) at 4 °C until the subsequent isolation procedure of the hACs^56^. Briefly, collected cartilage samples were dissected in small full-depth pieces, washed, and digested with 0.25% w/v trypsin solution (Sigma-Aldrich) for 30 min at 37 °C under agitation. Then the solution was removed, and the cartilage was washed and incubated overnight at 37 °C with a 2 mg mL^−1^ collagenase type II solution (Sigma-Aldrich). In the following day, the cells were washed, counted, and plated at a density of 2 × 10^6^ cells. Cells were expanded in expansion medium [EM; Dulbecco’s modified Eagle’s medium (Sigma-Aldrich) containing 10 mM Hepes buffer (Sigma-Aldrich), L-alanyl-L-glutamine (Sigma-Aldrich), non-essential amino acids (Sigma-Aldrich), 1% antibiotic–antimycotic solution, 10% fetal bovine serum (FBS), and 10 ng mL^−1^ basic fibroblast growth factor (bFGF; PeproTech)] at 37 °C in a humidified atmosphere of 5% CO_2_.

### Isolation and expansion of hBM-MSCs

Human bone marrow aspirates were collected, after obtaining informed consent from patient undergoing knee arthroplasty (female; aged 55), under the cooperation agreement established between the 3Bs Research Group, University of Minho and the Hospital Center of Alto Ave, Guimarães, Portugal. hBM-MSCs were isolated and characterized using our established standard protocols^57^. Cells were expanded in BM [MEM alpha medium (α-MEM; Sigma-Aldrich) supplemented with 10% heat-inactivated FBS (Life Technologies) and 1% antibiotic/antimycotic solution (final concentration of penicillin 100 units mL^−1^ and streptomycin 100 mg mL^−1^; Life Technologies)] at 37 °C in a humidified atmosphere of 5% CO_2_.

### Conditioned media

**Fig. 7:**
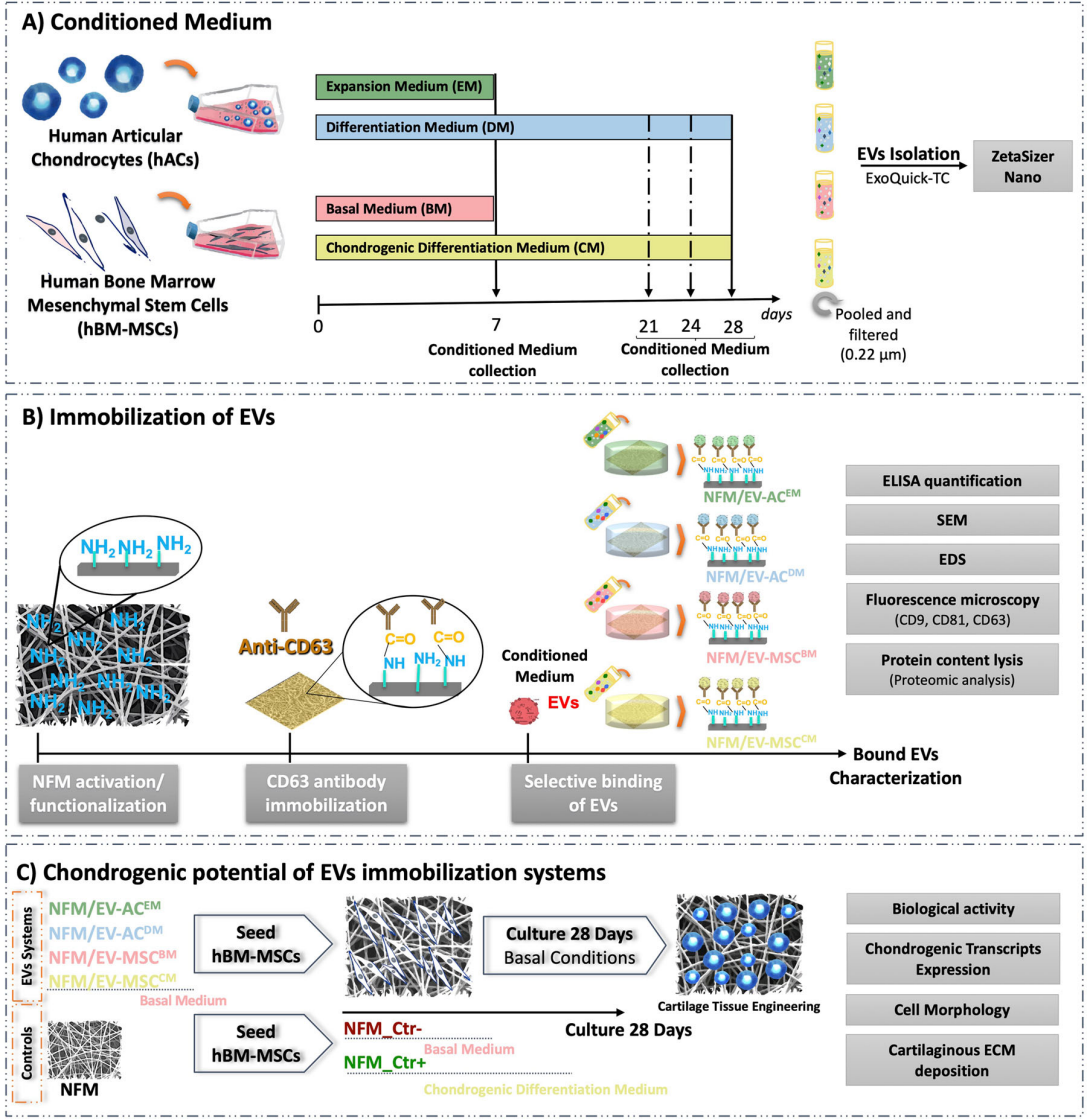
Schematic illustration of the experimental design. Conditioned media preparation (**A**); development of EV immobilization systems and characterization of bound EVs (**B**); assessment of the chondrogenic potential of EV immobilization systems (**C**).

Conditioned media harvested from the hACs and hBM-MSCs in culture were used as a source of EVs, according to the schematic illustration of the experimental design (Fig. 7). First, the hACs and hBM-MSCs were expanded at passage 3 and a cell suspension containing of 3 × 10^3^ cells cm^−2^ were subcultured in T150 flasks and cultured in EM and BM, respectively, at 37 °C in a humidified atmosphere of 5% CO_2_. The conditioned media were harvested when the cells reach the confluence (in 7 days), pooled, filtered (pore size 0.22 μm) to remove cell debris, and stored at −80 °C until further use. A cell suspension containing 2 × 10^5^ cells/15 mL centrifuge tubes of hACs or hBM-MSCs were also prepared to assure obtaining conditioned medium of chondrogenic lineage commitment. In order to form a spherical aggregate or pellet cultures, the cell aliquots were centrifuged at 600 × *g* for 5 min and incubated at 37 °C in a humidified atmosphere of 5% CO_2_ for 24 h incubation. The hACs and hBM-MSCs were cultures under DM [EM—instead of adding bFGF, 1 mg mL^−1^ L-ascorbic acid (Sigma-Aldrich) and 1 mg mL^−1^ insulin (Sigma-Aldrich) were added] or standard chondrogenic DM [CM; BM supplemented with Insulin-Transferrin-Selenium-G Supplement (ITS; Invitrogen), 1 mM dexamethasone (Sigma-Aldrich), 0.1 M sodium pyruvate (Invitrogen), 17 mM ascorbic acid-2-phosphate (Sigma-Aldrich), 35 mM L-proline (Sigma-Aldrich), and 10 ng mL^−1^ transforming growth factor-β3 (PeproTech)], respectively. Those conditioned media (DM; CM) were harvested at 21, 24, and 28 days of culture, pooled, filtered (pore size 0.22 μm) to remove cell debris, and stored at −80 °C until further use.

The amount of EVs presented on each conditioned medium was quantified by the Exosome ELISA Complete Kit (CD63) (System Biosciences) after EV isolation using a polymeric precipitation solution (ExoQuick-TC; System Biosciences, BioCat GmbH). Those assays were performed according to the manufacturer’s instructions.

The size of the isolated EVs was determined by dynamic light scattering at an angle of 173° and at a wavelength of 633 nm, and the zeta-potential was determined by laser Doppler electrophoresis using a Zetasizer Nano ZS instrument (Malvern Instruments).

### Preparation of activated and functionalized PCL NFMs

The production of electrospun PCL NFMs was performed as described in detail elsewhere^8^. In brief, a polymeric solution of 15% (w/v) PCL (Mn 70,000–90,000 by GPC, Sigma-Aldrich) in chloroform (Sigma-Aldrich) and *N*,*N*-dimethylformamide (7: 3 volume ratio; Sigma-Aldrich) was electrospun at 12 kV, using a needle-to-ground collector distance of 20 cm, and a flow rate of 1.0 mL h^−1^. The electrospun NFM is composed of nanofibers with diameters in the micrometer range, from 0.4 to 1.4 μm, with an average pore size of 7.267 ± 3.148 μm and a thickness range from 40 to 60 μm^58^.

Samples of electrospun PCL NFM (1 cm^2^) were activated in an ultraviolet−ozone system (ProCleaner 220, Bioforce Nanoscience) by exposing both sides for 2 min each. Incubation in 1 M 1,6-hexanediamine solution (Sigma-Aldrich) for 1 h at 37 °C was performed, in order to graft amine groups (-NH_2_) at the NFM surface.

### Engineered EV immobilization systems

Since EVs, namely, exosomes, typically express the CD63 surface marker, the human anti-CD63 antibody (E-12; Santa Cruz Biotechnology, Inc.) was immobilized at the surface of NFMs by a covalent bond mediated by a coupling agent, namely, 1-ethyl-3-(3-(dimethylamino)-propyl)carbodiimide/hydroxysuccinimide mixture (10 mM EDC + 40 mM NHS; Sigma-Aldrich, S.L.). The antibody solution (1% (v/v)) was mixed for 15 min at room temperature (RT), for the antibody activation, and incubated 2 h at RT on the activated and functionalized nanofibrous substrate.

The maximum immobilization capacity of the antibody over the nanofibrous substrate was determined by using a wide range of concentrations (from 0 to 8 μg mL^−1^). After the anti-CD63 antibody immobilization, a blocking step was performed by a 3% bovine serum albumin (BSA; Sigma) for 1 h at RT, followed by the secondary antibody (1:200 in PBS) incubation (1 h at RT). Alexa Fluor^®^ 488 rabbit anti-mouse (~495/517 nm; Life Technologies) was used as secondary antibody against the anti-CD63 antibody. The unbound secondary antibody fluorescence was measured in a microplate reader (Synergy HT, Bio-TEK), as an indirect method to determine the primary antibody immobilization efficiency. In order to evaluate nonspecific immobilization, the activated and functionalized NFM without primary antibody was used as a negative control. The samples were further analyzed by fluorescence microscopy (Axio Observer; Zeiss) to detect the distribution of the anti-CD63 antibody at the surface of the nanofibrous substrate.

Biofunctionalization of NFMs with EVs was achieved by using an antibody–antigen strategy, as described in detail elsewhere^59^. The nanofibrous substrate with immobilized anti-CD63 antibody, at the antibody concentration previously optimized, was incubated with the different conditioned medium previously harvested (i.e., AC^EM^, AC^DM^, MSC^BM^, MSC^CM^) for 2 h at RT. The unbound EV solutions were collected and quantified by enzyme-linked immunosorbent assay (ELISA; Exosome ELISA Complete Kit (CD63)), in order to define the binding capacity of the engineered EV immobilization system. The negative control samples (i.e., non-biofunctionalized NFM) were performed by carrying out all the biofunctionalization steps (including the incubation step with different conditioned media) but substituting the anti-CD63 antibody solution by the PBS solution.

The morphology of EVs bound at the surface of biofunctional electrospun PCL NFM (i.e., *NFM/EV-AC*^*EM*^, *NFM/EV-AC*^*DM*^, *NFM/EV-MSC*^*BM*^, *NFM/EV-MSC*^*CM*^) was analyzed by SEM (AURIGA Compact, Zeiss, Germany). By EDS (INCAx-Act, PentaFET Precision, Oxford Instruments), an elemental analysis of the EV immobilization systems was performed to further confirm the presence of the EVs at the surface of the NFMs.

The distribution of EVs bound to the NFM biofunctionalized with anti-CD63 antibody was performed by immunofluorescence staining. First, a blocking step (3% BSA for 30 min) was performed. In between each step, the samples were rinsed three times in PBS buffer. For EVs’ surface marker staining, samples were incubated with the primary antibodies CD63 (E-12; 1:500; Santa Cruz Biotechnology, Inc.), CD81 (1.3.3.22; 1:500; Santa Cruz Biotechnology, Inc.), and CD9 (C-4; 1:500; Santa Cruz Biotechnology, Inc.) overnight and then with the corresponding secondary antibody [Alexa Fluor^®^ 488 rabbit anti-mouse (~495/517 nm; Life Technologies)] for 1 h at RT. The non-biofunctionalized NFM was used as a negative control to evaluate nonspecific immunodetection of EVs’ surface markers in the biofunctionalized electrospun NFMs. The samples were further analyzed by fluorescence microscopy (Axio Observer; Zeiss).

### Proteomic analysis of the bound EVs

The total protein content of EVs bound to the surface of biofunctional NFM were prepared using RIPA buffer (Sigma) supplemented with protease inhibitor cocktail (Sigma) for 1 h at 4 °C. The samples were subjected to ultrasonic cracking, centrifuged for 15 min at 14,000 rpm at 4 °C, and the supernatants were collected.

Protein extracts of 15 μg were solubilized with 100 mM Tris pH 8.5, 1% sodium deoxycholate, 10 mM tris(2-carboxyethyl)phosphine and 40 mM chloroacetamide for 10 min at 95 °C at 1000 rpm (Thermomixer, Eppendorf). Each sample was processed for proteomic analysis following the solid-phase-enhanced sample-preparation (SP3) protocol as described in detail elsewhere^60^. Enzymatic digestion was performed with Trypsin/LysC overnight at 37 °C at 1000 rpm.

Protein identification and quantitation was performed by nanoLC-MS/MS as described in detail elsewhere^61^. The raw data was processed using the Proteome Discoverer 2.5.0.400 software (Thermo Scientific) and searched against the UniProt database for the *Homo sapiens* Proteome (2020_05 with 75069 entries), the NIST human spectral library, and *Bos taurus* Proteome (2020_05 with 37512 entries). A common protein contaminant list from MaxQuant was also considered in the analysis. The MSPepSearch and Sequest HT search engines were used to identify tryptic peptides. The ion mass tolerance was 10 ppm for precursor ions and 0.02 Da for fragmented ions in both softwares. Maximum allowed missing cleavage sites was set to two. Cysteine carbamidomethylation was defined as constant modification. Methionine oxidation, deamidation of glutamine and asparagine, peptide terminus glutamine to pyroglutamate, and protein N-terminus acetylation, Met-loss, and Met-loss+acetyl were defined as variable modifications. Peptide confidence was set to high. The processing node Percolator was enabled with the following settings: maximum delta Cn 0.05; decoy database search target false discovery rate 1%, validation based on *q*-value. Protein label-free quantitation was performed with the Minora feature detector node at the processing step. Precursor ions quantification was performed at the processing step with the following parameters: peptides to use all peptides, precursor abundance based on intensity, and normalization based on total peptide amount. Protein ratio was based on protein abundance and an analysis of variance (Individual Proteins) hypothesis test was performed.

### Bioinformatic analysis

The identified proteins were analyzed using the Venny tool (v2.1.0, https://bioinfogp.cnb.csic.es/tools/venny/). The sequences of the identified proteins were mapped according to their GO to determine their biological and functional properties, using InterProScan (v.5.14-53.0, http://www.ebi.ac.uk/interpro/). Proteins were grouped with regard to the biological process, cellular component, and molecular function using the categories of the Panther Biological process (v.15.0, http://www.pantherdb.org/). The GO analysis and heat map were obtained using the GraphPad PRISM v. 8.0. Clusters heat map were assembled by GO analysis using an Euclidean distance function.

### Cell seeding and culture conditions

The effectiveness of the developed EV immobilization systems, as chondrogenic lineage inducible systems, was assessed using hBM-MSCs (Table 1). Confluent hBM-MSCs at passage 4 were harvested for seeding on top of the EV immobilization systems (*NFM/EV-AC*^*EM*^, *NFM/EV-AC*^*DM*^, *NFM/EV-MSC*^*BM*^, *NFM/EV-MSC*^*CM*^) at a density of 2 × 10^5^ cells per sample. These constructs were cultured under BM, without further medium supplementation. The experimental control conditions comprise hBM-MSCs cultured on top of the non-biofunctionalized NFMs under BM (*NFM_Ctrl−*) and using the standard chondrogenic DM (*NFM_Ctrl*+). hACs were also seeded on top of the non-biofunctionalized NFMs at a density of 2 × 10^5^ cells per sample and cultured under EM (*NFM_EM*) or DM (*NFM_DM*) (Supporting Information section—Supplementary Figs. 4 and 5). The constructs were retrieved for further analysis at predefined culturing times, namely, 0, 7, 14, 21, and 28 days. All experiments were performed in triplicate and repeated at least three times (*n* = 3), independently.

**Table 1:** Experimental conditions used in the cell biology assays.

Condition	Description	Cells	Medium
NFM_Ctrl−	Non-biofunctionalized NFMs under BM	hBM-MSCs	BM
NFM_Ctrl+	Non-biofunctionalized NFMs under CM		CM
NFM/EV-AC^EM^	Biofunctional NFMs with EVs derived from hACs under EM		BM
NFM/EV-AC^DM^	Biofunctional NFMs with EVs derived from hACs under DM		
NFM/EV-MSC^BM^	Biofunctional NFMs with EVs derived from hBM-MSCs under BM		
NFM/EV-MSC^CM^	Biofunctional NFMs with EVs derived from hBM-MSCs under CM		
NFM_EM	Non-biofunctionalized NFMs under EM	hACs	EM
NFM_DM	Non-biofunctionalized NFMs under DM		DM

*EM* expansion medium, *DM* differentiation medium, *BM* basal medium, *CM* chondrogenic medium.

### Cellular biochemistry analysis

The metabolism was evaluated by the MTS assay (CellTiter 96 AQ_ueous_ One Solution, Promega), the cell proliferation by DNA quantification (Quant-iTPicoGreen dsDNA assay, Invitrogen, Alfagene), and the protein synthesis by the Micro BCA assay (Micro BCA^TM^ Protein Assay Kit, Thermo Fisher Scientific), according to the manufacturers’ instructions. GAG quantification was performed according to our established standard colorimetric assay^8^.

### Scanning electron microscopy

The constructs were collected after 28 days of culture and fixed with 2.5% glutaraldehyde. By increasing the alcohol concentrations, samples were dehydrated, followed by sputter coating with Au/Pd. A scanning electron microscope (JSM-6010 LV, JEOL, Japan) was used to observe the distribution and morphology of the cells at ×3000 magnification.

### Gene expression analysis

At each time point, the constructs were washed, immersed in Tri reagent^®^ (Life Science, VWR), and kept at −80 °C. Total RNA was isolated and reverse transcribed into cDNA (qScript cDNA synthesis kit, Quanta Biosciences), followed by quantitative polymerase chain reaction (qPCR; PerfeCta^TM^ SYBR^®^ Green system; Quanta Biosciences), according to the manufacturer’s instructions. The qPCR reactions were carried out in a Mastercycler^®^ ep Gradient S realplex^®^ thermocycler (Eppendorf; Hamburg) for the target genes described in Table 2. The transcript expression data were normalized against the housekeeping gene *Glyceraldehyde 3-phosphate dehydrogenase* and the quantification was performed according to the Livak method (2^−ΔΔCT^ method). For hBM-MSC samples, the BM condition (*NFM_Ctrl-*) was used as calibrator, while the EM condition (*NFM_EM*) was used as calibrator of the samples of hACs.

**Table 2:** Primer sequences used for RT-PCR procedures.

	Gene	Forward (5′− 3′)	Reverse (5′−3′)
Ref.	*GAPDH*	AGCCTCAAGATCATCAGCAA	GTCATGAGTCCTTCCACGAT
Chondrogenic	*Sox9*	TTCATGAAGATGACCGACGC	GTCCAGTCGTAGCCCTTGAG
	*Aggrecan*	TGAGTCCTCAAGCCTCCTGT	TGGTCTGCAGCAGTTGATTC
	*COMP*	AGGGATGGAGACGGACATCAG	TCTGCATCAAAGTCGTCCTG
	*COL II*	CGGTGAGAAGGGAGAAGTTG	GACCGGTCACTCCAGTAGGA
Hypertrophic	*COL Iα*	AAGAACCCCAAGGACAAGAG	GTAGGTGATGTTCTGGGAGG
	*COL X*	CAGGCATAAAAGGCCCACTA	AGGACTTCCGTAGCCTGGTT

*GAPDH* glyceraldehyde 3-phosphate dehydrogenase, *Sox9* Sry-type high mobility group box 9, *COMP* cartilage oligomeric matrix protein, *COL II* collagen type II, *COL Iα* collagen type I alpha, *COL X* collagen type X.

### Histological analysis

Constructs were collected after 28 days of culture, fixed in a 4% paraformaldehyde solution, and kept at 4 °C until further used. The staining procedures were performed on top of the samples. Alcian blue staining, were performed as described elsewhere^62^. For immunohistochemistry, the samples were permeabilized using 0.1% Triton X-100 in PBS for 15 min, incubated with 3% BSA, and incubated with a defined primary antibody (collagens type I (COL1A1, clone C-18; Santa Cruz Biotechnology) and type II (mouse anti-human type II collagen monoclonal antibody; Millipore); actin (mouse anti-alpha smooth muscle Actin antibody; Abcam)) overnight at 4 °C. The samples were then washed with PBS and stained with the corresponding secondary antibodies (Life Technologies). Nuclei were counter-stained with 4′,6-diamidino-2-phenylindole (1 mg mL^−1^ in PBS for 15 min). Fluorescence images from stained constructs were obtained using a confocal laser scanning microscope (Leica TCS SP8).

### Statistical analysis

Statistical analysis was performed using the SPSS statistic software (release 24.0.0.0 for Mac). First, Shapiro–Wilk test was used to ascertain the data normality and Levene test for the homogeneity of variances. Observing this, the normality and variance homogeneity were rejected; non-parametric tests were used (Kruskal–Wallis test followed by Tukey’s Honest Significant Difference test). The confidence interval used was 99% and *p* ≤ 0.01 were regarded as statistically significant.

### Ethical approval

All procedures performed in studies involving human participants were in accordance with the ethical standards of the University of Minho Life Sciences Ethics Committee (SECVS 136/2015), the Hospital Center of Alto Ave, Guimarães, Portugal (67/CA), and the 1964 Helsinki Declaration and its later amendments or comparable ethical standards.

### Reporting summary

Further information on research design is available in the Nature Research Reporting Summary linked to this article.

## Supplementary information

Supplementary Information

Reporting Summary

## Data Availability

The data that support the findings of this study are available from the corresponding author upon reasonable request.

## References

[1] Urbanek O, Kolbuk D, Wrobel M (2019). Articular cartilage: new directions and barriers of scaffolds development - review. Int J. Polym. Mater. Polym. Biomater..

[2] Mao AS, Mooney DJ (2015). Regenerative medicine: current therapies and future directions. Proc. Natl Acad. Sci. USA.

[3] Armoiry X (2019). Autologous chondrocyte implantation with chondrosphere for treating articular cartilage defects in the knee: an evidence review group perspective of a NICE Single Technology Appraisal. Pharmacoeconomics.

[4] Choi S (2018). Autologous bone marrow cell stimulation and allogenic chondrocyte implantation for the repair of full-thickness articular cartilage defects in a rabbit model. Cartilage.

[5] Behrens P, Bitter T, Kurz B, Russlies M (2006). Matrix-associated autologous chondrocyte transplantation/implantation (MACT/MACI) - 5-year follow-up. Knee.

[6] Akgun I (2015). Matrix-induced autologous mesenchymal stem cell implantation versus matrix-induced autologous chondrocyte implantation in the treatment of chondral defects of the knee: a 2-year randomized study. Arch. Orthop. Trauma Surg..

[7] Longoni A (2018). The impact of immune response on endochondral bone regeneration. Npj Regen. Med..

[8] Casanova MR (2019). Chondrogenesis-inductive nanofibrous substrate using both biological fluids and mesenchymal stem cells from an autologous source. Mat. Sci. Eng. C. Mater..

[9] Kaul G (2006). Local stimulation of articular cartilage repair by transplantation of encapsulated chondrocytes overexpressing human fibroblast growth factor 2 (FGF-2) in vivo. J. Gene Med..

[10] Pereira RC (2009). Novel injectable gel (system) as a vehicle for human articular chondrocytes in cartilage tissue regeneration. J. Tissue Eng. Regen. Med..

[11] Dickhut A (2009). Calcification or dedifferentiation: requirement to lock mesenchymal stem cells in a desired differentiation stage. J. Cell Physiol..

[12] Graceffa V (2019). Chasing chimeras - the elusive stable chondrogenic phenotype. Biomaterials.

[13] Casanova MR, Reis RL, Martins A, Neves NM (2020). Fibronectin bound to a fibrous substrate has chondrogenic induction properties. Biomacromolecules.

[14] Maas SLN, Breakefield XO, Weaver AM (2017). Extracellular vesicles: unique intercellular delivery vehicles. Trends Cell Biol..

[15] Raposo G, Stoorvogel W (2013). Extracellular vesicles: exosomes, microvesicles, and friends. J. Cell Biol..

[16] Ji YH (2019). Multiplexed profiling of single-cell extracellular vesicles secretion. Proc. Natl Acad. Sci. USA.

[17] Yeh YT (2020). Rapid size-based isolation of extracellular vesicles by three-dimensional carbon nanotube arrays. Acs Appl. Mater. Interfaces.

[18] Cunnane EM (2020). Extracellular vesicles enhance the remodeling of cell-free silk vascular scaffolds in rat aortae. ACS Appl. Mater. Interfaces.

[19] Witwer, K. W. et al. Standardization of sample collection, isolation and analysis methods in extracellular vesicle research. *J. Extracell. Vesicles*10.3402/jev.v2i0.20360 (2013). 10.3402/jev.v2i0.20360 PMC3760646 24009894

[20] Thery C (2018). Minimal information for studies of extracellular vesicles 2018 (MISEV2018): a position statement of the International Society for Extracellular Vesicles and update of the MISEV2014 guidelines. J. Extracell. Vesicles.

[21] Bjorge IM, Kim SY, Mano JF, Kalionis B, Chrzanowski W (2017). Extracellular vesicles, exosomes and shedding vesicles in regenerative medicine - a new paradigm for tissue repair. Biomater. Sci..

[22] Jay SM, Vunjak-Novakovic G (2017). Extracellular vesicles and their versatile roles in tissue engineering. Tissue Eng. Pt. A.

[23] Lamichhane TN (2015). Emerging roles for extracellular vesicles in tissue engineering and regenerative medicine. Tissue Eng. Pt. B Rev..

[24] Bian SY (2014). Extracellular vesicles derived from human bone marrow mesenchymal stem cells promote angiogenesis in a rat myocardial infarction model. J. Mol. Med..

[25] Bruno, S. et al. Microvesicles derived from human bone marrow mesenchymal stem cells inhibit tumor growth. *Stem Cells Dev*. 10.1089/scd.2012.0304 (2013). 10.1089/scd.2012.0304 23034046

[26] Martins M, Ribeiro D, Martins A, Reis RL, Neves NM (2016). Extracellular vesicles derived from osteogenically induced human bone marrow mesenchymal stem cells can modulate lineage commitment. Stem Cell Rep..

[27] Ruiz M (2020). TGFBI secreted by mesenchymal stromal cells ameliorates osteoarthritis and is detected in extracellular vesicles. Biomaterials.

[28] Zhang SP (2019). MSC exosomes alleviate temporomandibular joint osteoarthritis by attenuating inflammation and restoring matrix homeostasis. Biomaterials.

[29] Malda J, Boere J, van de Lest CHA, van Weeren PR, Wauben AHM (2016). Extracellular vesicles - new tool for joint repair and regeneration. Nat. Rev. Rheumatol..

[30] Kim M, Steinberg DR, Burdick JA, Mauck RL (2019). Extracellular vesicles mediate improved functional outcomes in engineered cartilage produced from MSC/chondrocyte cocultures. Proc. Natl Acad. Sci. USA.

[31] Roccio M (2013). Predicting stem cell fate changes by differential cell cycle progression patterns. Development.

[32] Kristensen AR, Gsponer J, Foster LJ (2013). Protein synthesis rate is the predominant regulator of protein expression during differentiation. Mol. Syst. Biol..

[33] Berckmans RJ (2002). Cell-derived microparticles in synovial fluid from inflamed arthritic joints support coagulation exclusively via a factor VII-dependent mechanism. Arthritis Rheum..

[34] Rani S, Ryan AE, Griffin MD, Ritter T (2015). Mesenchymal stem cell-derived extracellular vesicles: toward cell-free therapeutic applications. Mol. Ther..

[35] Buzas EI, Gyorgy B, Nagy G, Falus A, Gay S (2014). Emerging role of extracellular vesicles in inflammatory diseases. Nat. Rev. Rheumatol..

[36] Lai RC (2010). Exosome secreted by MSC reduces myocardial ischemia/reperfusion injury. Stem Cell Res..

[37] Zhou Y (2013). Exosomes released by human umbilical cord mesenchymal stem cells protect against cisplatin-induced renal oxidative stress and apoptosis in vivo and in vitro. Stem Cell Res. Ther..

[38] Zhang HC (2012). Microvesicles derived from human umbilical cord mesenchymal stem cells stimulated by hypoxia promote angiogenesis both in vitro and in vivo. Stem Cells Dev..

[39] Zhang B (2014). Mesenchymal stem cells secrete immunologically active exosomes. Stem Cells Dev..

[40] Lee C (2012). Exosomes mediate the cytoprotective action of mesenchymal stromal cells on hypoxia-induced pulmonary hypertension. Circulation.

[41] Kordelas L (2014). MSC-derived exosomes: a novel tool to treat therapy-refractory graft-versus-host disease. Leukemia.

[42] Stewart K (2003). STRO-1, HOP-26 (CD63), CD49a and SB-10 (CD166) as markers of primitive human marrow stromal cells and their more differentiated progeny: a comparative investigation in vitro. Cell Tissue Res..

[43] Lee HJ, Choi BH, Min BH, Park SR (2009). Changes in surface markers of human mesenchymal stem cells during the chondrogenic differentiation and dedifferentiation processes in vitro. Arthritis Rheum..

[44] Chen TS (2010). Mesenchymal stem cell secretes microparticles enriched in pre-microRNAs. Nucleic Acids Res..

[45] Tan, S. S. et al. Therapeutic MSC exosomes are derived from lipid raft microdomains in the plasma membrane. *J. Extracell. Vesicles*. 10.3402/jev.v2i0.22614 (2013). 10.3402/jev.v2i0.22614 PMC3873122 24371518

[46] Wang C (2016). Sox9-induced chondrogenesis in mesenchymal stem cells was mediated by ERK5 signal pathway. Cell Mol. Biol..

[47] Pan QH (2008). Sox9, a key transcription factor of bone morphogenetic protein-2-induced chondrogenesis, is activated through BMP pathway and a CCAAT box in the proximal promoter. J. Cell Physiol..

[48] Hardingham TE, Oldershaw RA, Tew SR (2006). Cartilage, SOX9 and notch signals in chondrogenesis. J. Anat..

[49] Gutierrez ML, Guevara JM, Echeverri OY, Garzon-Alvarado D, Barrera LA (2013). Aggrecan catabolism during mesenchymal stromal cell in vitro chondrogenesis. Anim. Cells Syst..

[50] Mitton E, Gohr CM, McNally MT, Rosenthal AK (2009). Articular cartilage vesicles contain RNA. Biochem. Biophys. Res. Commun..

[51] Derfus BA (2001). Transforming growth factor beta-1 stimulates articular chondrocyte elaboration of matrix vesicles capable of greater calcium pyrophosphate precipitation. Osteoarthr. Cartil..

[52] Rosenthal AK, Gohr CM, Ninomiya J, Wakim BT (2011). Proteomic analysis of articular cartilage vesicles from normal and osteoarthritic cartilage. Arthritis Rheum..

[53] Zhang M, Schekman R (2013). Cell biology. Unconventional secretion, unconventional solutions. Science.

[54] Rosenthal AK (2015). Autophagy modulates articular cartilage vesicle formation in primary articular chondrocytes. J. Biol. Chem..

[55] Casanova MR, Reis RL, Martins A, Neves NM (2020). Surface biofunctionalization to improve the efficacy of biomaterial substrates to be used in regenerative medicine. Mater. Horiz..

[56] da Silva MLA (2015). Conditioned medium as a strategy for human stem cells chondrogenic differentiation. J. Tissue Eng. Regen. Med..

[57] da Silva MLA (2010). Cartilage tissue engineering using electrospun PCL nanofiber meshes and MSCs. Biomacromolecules.

[58] da Silva MA (2009). Evaluation of extracellular matrix formation in polycaprolactone and starch-compounded polycaprolactone nanofiber meshes when seeded with bovine articular chondrocytes. Tissue Eng. Pt. A.

[59] Oliveira C, Costa-Pinto AR, Reis RL, Martins A, Neves NM (2014). Biofunctional anofibrous substrate comprising immobilized antibodies and selective binding of autologous growth factors. Biomacromolecules.

[60] Hughes CS (2019). Single-pot, solid-phase-enhanced sample preparation for proteomics experiments. Nat. Protoc..

[61] Osorio, H. et al. Proteomics analysis of gastric cancer patients with diabetes mellitus. *J. Clin. Med*. 10.3390/jcm10030407 (2021). 10.3390/jcm10030407 PMC7866049 33494396

[62] da Silva MA, Martins A, Teixeira AA, Reis RL, Neves NM (2010). Impact of biological agents and tissue engineering approaches on the treatment of rheumatic diseases. Tissue Eng. Pt. B Rev..

