# Author Correction: Chondrogenic differentiation induced by extracellular vesicles bound to a nanofibrous substrate

**DOI:** 10.1038/s41536-023-00300-8

**Published:** 2023-05-22

**Authors:** Marta R. Casanova, Hugo Osório, Rui L. Reis, Albino Martins, Nuno M. Neves

**Affiliations:** 1 3B’s Research Group, I3Bs - Research Institute on Biomaterials, Biodegradables and Biomimetics of University of Minho, Headquarters of the European Institute of Excellence on Tissue Engineering and Regenerative Medicine, AvePark – Parque de Ciência e Tecnologia, Zona Industrial da Gandra, 4805-017 Barco/Guimarães, Portugal; 2 ICVS/3B’s – PT Government Associate Laboratory, Braga/Guimarães, Portugal; 3 i3S — Instituto de Investigação e Inovação em Saúde, Universidade do Porto, 4200-135 Porto, Portugal; 4 Ipatimup—Institute of Molecular Pathology and Immunology of the University of Porto, University of Porto, 4200-135 Porto, Portugal; 5 Department of Pathology, Faculty of Medicine, University of Porto, 4200-319 Porto, Portugal

**Keywords:** Stem-cell differentiation, Biomaterials - cells, Mesenchymal stem cells

Correction to: *npj Regenerative Medicine* 10.1038/s41536-021-00190-8, published online 19 November 2021

In the Acknowledgements section, the funding source ‘project Cells4_IDs (PTDC/BTM-SAL/28882/2017)’ should have read as ‘project POCI-01-0145-FEDER-028882 (PTDC/BTM-SAL/28882/2017) co-financiado pelo Fundo Europeu de Desenvolvimento Regional - FEDER, através do Programa Operacional Competitividade e Internacionalização e pelo Orçamento de Estado, através da FCT.’. The original article has been corrected.

