# Supplementary material for: Chondrogenic differentiation induced by extracellular vesicles bound to a nanofibrous substrate
Source: NPJ Regen Med. 2021 Nov 19;6:79. doi: 10.1038/s41536-021-00190-8 (PMC8604977; doi:10.1038/s41536-021-00190-8)
Supplement: Supplementary file 1 — Supplementary Information [file 41536_2021_190_MOESM1_ESM.pdf]

SUPPORTING INFORMATION

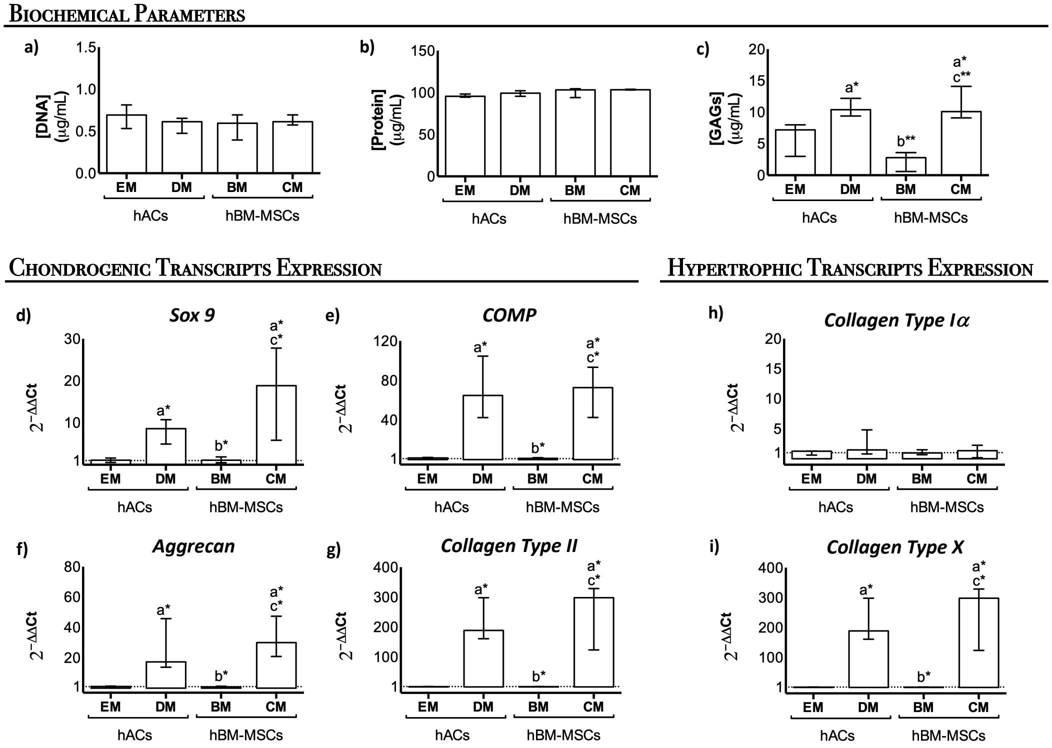

Supplementary Figure 1. Biochemical performance (i.e. proliferation (DNA content) (**a**), total protein synthesis(**b**)), sulfated glycosaminoglycans (GAGs) content (**c**) and relative expression of chondrogenic (i.e. *Sox 9* (**d**), *COMP* (**e**), *Aggrecan* (**f**) and *Collagen type II* (**g**)) and hypertrophic transcripts (i.e. *Collagen type Iα* (**h**), *Collagen type X* (**i**)) by hACs cultured on non-biofunctionalized nanofibrous substrates under expansion medium (*EM*) or differentiation medium (*DM*), and hBM-MSCs cultured on non-biofunctionalized nanofibrous substrates under basal medium (*BM*) or chondrogenic medium (*CM*). Data were analyzed by the Kruskal-Wallis test, followed by the Tukey's HSD test ($^{*}p$<0.01; $^{**}p$<0.001): *a* denotes significant differences compared to *EM*; *b* denotes significant differences compared to *DM*; *c* denotes significant differences compared to *BM*.

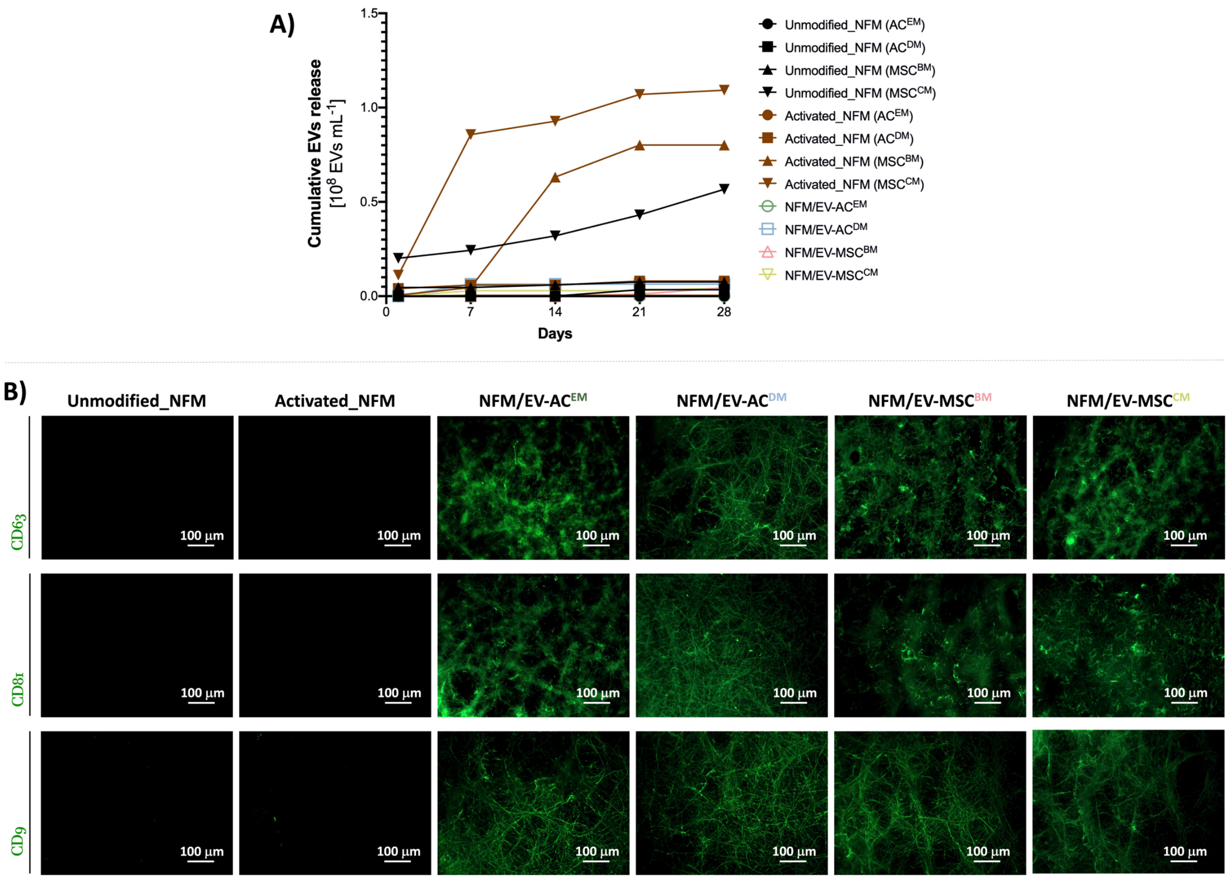

Supplementary Figure 2. EVs' release study from biofunctional nanofibrous systems comprising EVs derived from different sources (i.e. NFM/EV-AC^{EM}, NFM/EV-AC^{DM}, NFM/EV-MSC^{BM} and NFM/EV-MSC^{CM}), under basal culture conditions during 28 days. The unmodified and activated NFM incubated with different conditioned media were also carried out. A) Cumulative EVs release; B) Fluorescence micrographs of the EVs markers (CD63, CD81 and CD9) after 28 days of culture.

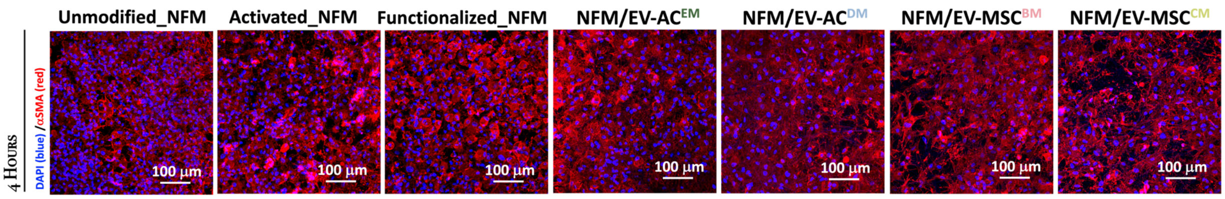

Supplementary Figure 3. Effect of the unmodified, activated or functionalized NFM and the biofunctional nanofibrous systems comprising EVs derived from different sources (*i.e.* NFM/EV-AC^{EM}, NFM/EV-AC^{DM}, NFM/EV-MSC^{BM} and NFM/EV-MSC^{CM}) on the behavior of hBM-MSCs demonstrated by immunohistochemistry (red for actin (αSMA) and blue for nuclei).

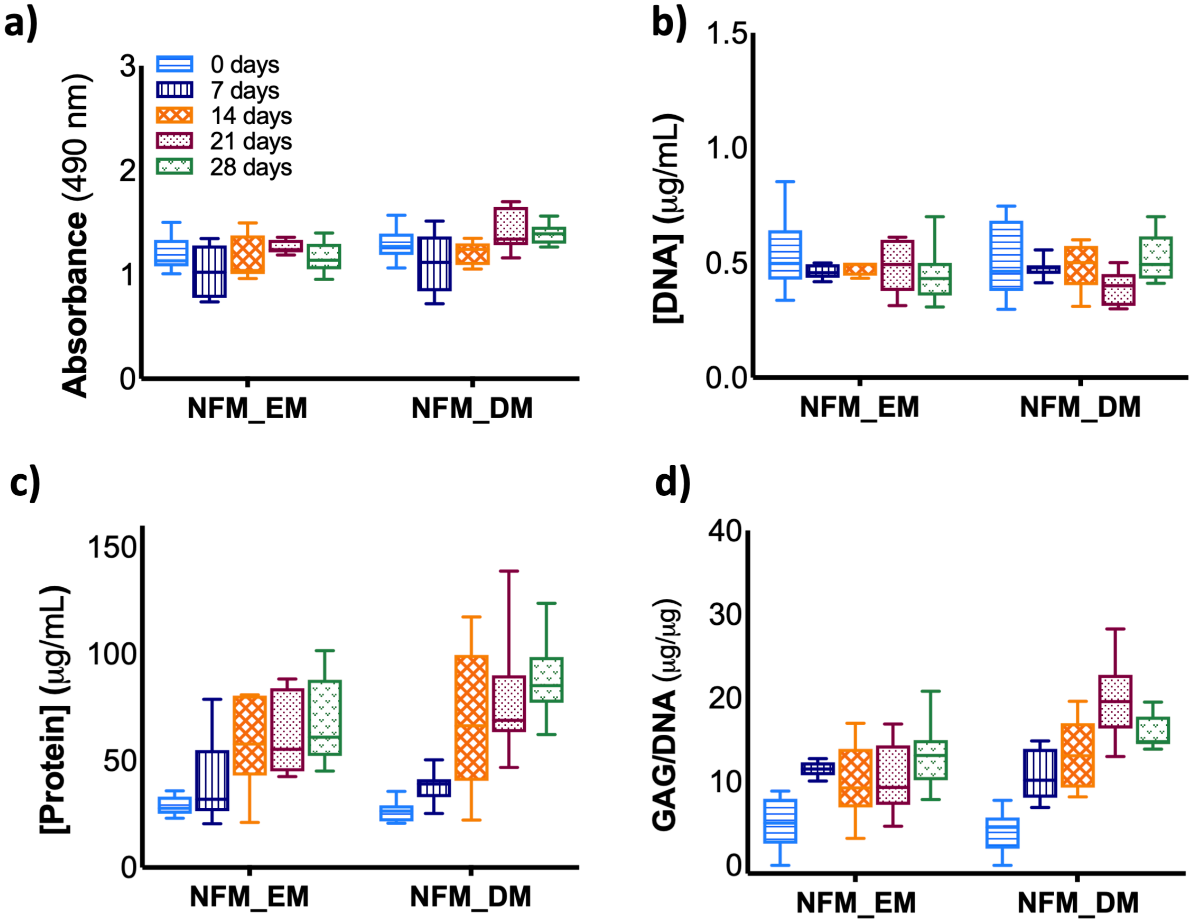

Supplementary Figure 4. Biochemical performance (i.e. cell viability (**a**), proliferation (DNA content) (**b**), total protein synthesis(**c**)) and sulfated glycosaminoglycans (GAGs) content normalized against DNA (**d**) of hACs cultured on non-biofunctionalized nanofibrous substrates under expansion medium (*NFM_EM*) or differentiation medium (*NFM_DM*). Data were analyzed by the Kruskal-Wallis test, followed by the Tukey's HSD test (p<0.01): no significant differences where observed between culturing conditions.

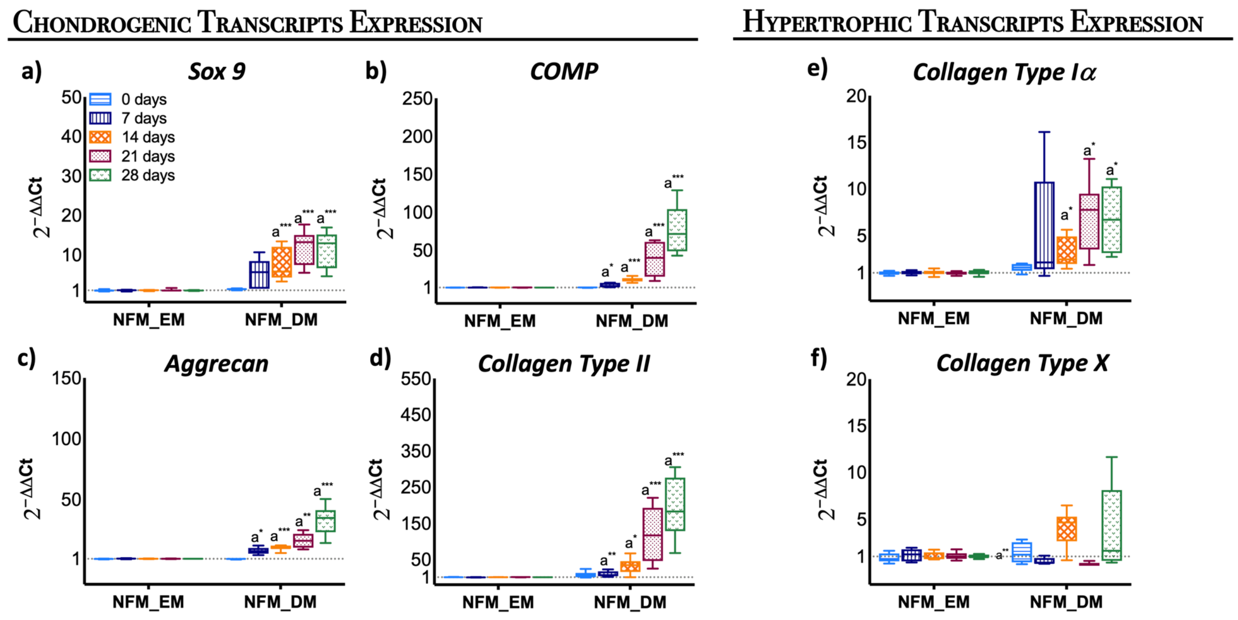

Supplementary Figure 5. Relative expression of chondrogenic (i.e. *Sox 9* (**a**), *COMP* (**b**), *Aggrecan* (**c**) and *Collagen type II* (**d**)) and hypertrophic transcripts (i.e. *Collagen type Iα* (**e**), *Collagen type X* (**f**)) by hACs cultured on non-biofunctionalized nanofibrous substrates under expansion medium (*NFM_EM*) or differentiation medium (*NFM_DM*). Data were analyzed by the Kruskal-Wallis test, followed by the Tukey's HSD test ($^{*}p$<0.01; $^{**}p$<0.001; $^{***}p$<0.0001): *a* denotes significant differences compared to *NFM_EM*.

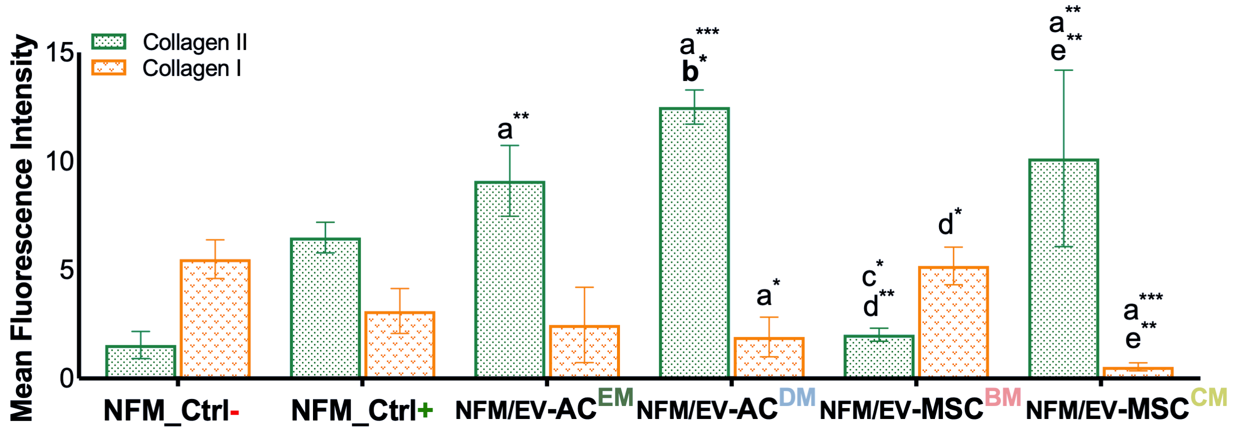

Supplementary Figure 6. Quantification of mean fluorescence intensity of fluorescence micrographs of collagen II and I are represented in Figure 7.

Supplementary Table 1. Zeta potential, diameter and PDI values obtained by DLS and laser Doppler electrophoresis, respectively, for extracellular vesicles (EVs) isolated from the different conditioned media harvested from hACs cultured under expansion (AC^{EM}) or differentiation (AC^{DM}) media, as well as from hBM-MSCs cultured under basal (MSC^{BM}) or chondrogenic (MSC^{CM}) media.

**EVs**	**Zeta potential** (mV)	**Size** (nm)	**PDI**
AC^{EM}	-7.8 ± 0.7	95 ± 1	0.279 ± 0.008
AC^{DM}	-9.3 ± 1.1	104 ± 13	0.282 ± 0.020
MSC^{BM}	-8.8 ± 1.0	97 ± 11	0.402 ± 0.006
MSC^{CM}	-7.4 ± 0.4	105 ± 12	0.423 ± 0.002
