# Supplementary material for: Chondrogenic differentiation induced by extracellular vesicles bound to a nanofibrous substrate
Source: NPJ Regen Med. 2021 Nov 19;6:79. doi: 10.1038/s41536-021-00190-8 (PMC8604977; doi:10.1038/s41536-021-00190-8)
Supplement: Supplementary file 2 — Reporting Summary [file 41536_2021_190_MOESM2_ESM.pdf]

nature portfolio

Corresponding author(s): Nuno M. Neves

Last updated by author(s): Sep 20, 2021

Reporting Summary

Nature Portfolio wishes to improve the reproducibility of the work that we publish. This form provides structure for consistency and transparency in reporting. For further information on Nature Portfolio policies, see our Editorial Policies and the Editorial Policy Checklist.

Statistics

For all statistical analyses, confirm that the following items are present in the figure legend, table legend, main text, or Methods section.

n/a	Confirmed	
☐	☒	The exact sample size (n) for each experimental group/condition, given as a discrete number and unit of measurement
☐	☒	A statement on whether measurements were taken from distinct samples or whether the same sample was measured repeatedly
☐	☒	The statistical test(s) used AND whether they are one- or two-sided<br>*Only common tests should be described solely by name; describe more complex techniques in the Methods section.*
☐	☒	A description of all covariates tested
☐	☒	A description of any assumptions or corrections, such as tests of normality and adjustment for multiple comparisons
☐	☒	A full description of the statistical parameters including central tendency (e.g. means) or other basic estimates (e.g. regression coefficient) AND variation (e.g. standard deviation) or associated estimates of uncertainty (e.g. confidence intervals)
☐	☒	For null hypothesis testing, the test statistic (e.g. F, t, r) with confidence intervals, effect sizes, degrees of freedom and P value noted<br>*Give P values as exact values whenever suitable.*
☒	☐	For Bayesian analysis, information on the choice of priors and Markov chain Monte Carlo settings
☒	☐	For hierarchical and complex designs, identification of the appropriate level for tests and full reporting of outcomes
☒	☐	Estimates of effect sizes (e.g. Cohen's d, Pearson's r), indicating how they were calculated

Our web collection on statistics for biologists contains articles on many of the points above.

Software and code

Policy information about availability of computer code

Data collection	*Provide a description of all commercial, open source and custom code used to collect the data in this study, specifying the version used OR state that no software was used.*
Data analysis	*Provide a description of all commercial, open source and custom code used to analyse the data in this study, specifying the version used OR state that no software was used.*

For manuscripts utilizing custom algorithms or software that are central to the research but not yet described in published literature, software must be made available to editors and reviewers. We strongly encourage code deposition in a community repository (e.g. GitHub). See the Nature Portfolio guidelines for submitting code & software for further information.

Data

Policy information about availability of data

All manuscripts must include a data availability statement. This statement should provide the following information, where applicable:

- Accession codes, unique identifiers, or web links for publicly available datasets
- A description of any restrictions on data availability
- For clinical datasets or third party data, please ensure that the statement adheres to our policy

The datasets generated during and/or analysed during the current study are available from the corresponding author on reasonable request.

Field-specific reporting

Please select the one below that is the best fit for your research. If you are not sure, read the appropriate sections before making your selection.

☒ Life sciences ☐ Behavioural & social sciences ☐ Ecological, evolutionary & environmental sciences

For a reference copy of the document with all sections, see nature.com/documents/nr-reporting-summary-flat.pdf

Life sciences study design

All studies must disclose on these points even when the disclosure is negative.

Sample size	The samples were tested in triplicates, in three independent assays.
Data exclusions	Significant outliers were excluded.
Replication	All assays were conducted three times, independently.
Randomization	Randomization was not relevant to this type of study.
Blinding	Blinding was not important to this study because only one sample of human chondrocytes or one sample of human bone marrow mesenchymal stem cells were used.

Reporting for specific materials, systems and methods

We require information from authors about some types of materials, experimental systems and methods used in many studies. Here, indicate whether each material, system or method listed is relevant to your study. If you are not sure if a list item applies to your research, read the appropriate section before selecting a response.

Materials & experimental systems

n/a	Involved in the study	
☐	☒	Antibodies
☒	☐	Eukaryotic cell lines
☒	☐	Palaeontology and archaeology
☒	☐	Animals and other organisms
☐	☒	Human research participants
☒	☐	Clinical data
☒	☐	Dual use research of concern

Methods

n/a	Involved in the study	
☒	☐	ChIP-seq
☒	☐	Flow cytometry
☒	☐	MRI-based neuroimaging

Antibodies

Antibodies used	anti-CD63 antibody (E-12; Santa Cruz Biotechnology, Inc.); anti-CD81 antibody (1.3.3.22; Santa Cruz Biotechnology, Inc.);; anti-CD9 antibody (C-4; Santa Cruz Biotechnology, Inc.); anti-collagen type I antibody (COL1A1, clone C-18; Santa Cruz Biotechnology); anti-collagen type II (mouse anti-human type II collagen monoclonal antibody, Millipore); anti-actin antibody (mouse anti-alpha smooth muscle Actin antibody; Abcam)
Validation	anti-CD63 antibody (mouse anti-human for IP, WB, IHC(P) and ELISA);<br>anti-CD81 antibody (mouse anti-human for WB, FCM, IF, IHC-p, IP);<br>anti-CD9 antibody (mouse anti-human for WB, IP, IF, IHC(P), FCM and ELISA);<br>anti-collagen type I antibody (mouse anti-human for WB, IP, IF, IHC(P) and ELISA);<br>anti-collagen type II (mouse anti-human for IF, IH & WB);<br>anti-actin antibody (mouse anti-human for WB, IHC/ICC, IP & Flow).

Human research participants

Policy information about studies involving human research participants

Population characteristics	No population characteristics were studied.
Recruitment	Human samples were collected under Informed Consent from patients undergoing knee arthroplasty.
Ethics oversight	Hospital Center of Alto Ave, Guimarães, Portugal.

Note that full information on the approval of the study protocol must also be provided in the manuscript.
